# B-CD8^+^ T Cell Interactions in the Anti-Idiotypic Response against a Self-Antibody

**DOI:** 10.1155/2017/2860867

**Published:** 2017-04-09

**Authors:** Darel Martínez, Amaury Pupo, Lianet Cabrera, Judith Raymond, Nichol E. Holodick, Ana María Hernández

**Affiliations:** ^1^Tumor Immunology Direction, Center of Molecular Immunology, Havana, Cuba; ^2^Systems Biology Direction, Center of Molecular Immunology, Havana, Cuba; ^3^Immunobiology Laboratory, Center for Oncology and Cell Biology, The Feinstein Institute for Medical Research, New York, NY, USA

## Abstract

P3 is a murine, germline, IgM mAb that recognizes *N*-glycolylated gangliosides and other self-antigens. This antibody is able to induce an anti-idiotypic IgG response and B-T idiotypic cascade, even in the absence of any adjuvant or carrier protein. P3 mAb immunization induces the expression of activation markers in a significant percentage of B-1a cells in vivo. Interestingly, transfer of both B-1a and B-2 to BALB/Xid mice was required to recover anti-P3 IgG response in this model. In fact, P3 mAb activated B-2 cells, in vitro, inducing secretion of IFN-γ and IL-4, although this activation was not detected ex vivo. Interestingly, naïve CD8^+^ T cells increased the expression of activation markers and IFN-γ secretion in the presence of B-1a cells isolated from P3 mAb-immunized mice, even without in vitro restimulation. In contrast, B-2 cells were able to stimulate CD8^+^ T cells only if P3 was added in vitro. Using bioinformatics, a MHC class I-binding peptide from P3 V_H_ region was identified. P3 mAb was able to induce a specific CTL response in vivo against cells presenting this peptide. Both humoral and CTL anti-idiotypic responses could be mechanisms to protect against the self-reactive antibody, contributing to keeping the tolerance to self-antigens.

## 1. Introduction

P3 is a monoclonal antibody (mAb) of IgM isotype, which recognizes *N*-glycolylated gangliosides and sulfatides, both self-antigens in mice [1]. It was also reported that the variable region of P3 is shared by the antibody A4ac, isolated from a mouse with experimental allergic encephalomyelitis, which recognizes myelin oligodendrocytes [2]. The V_H_ of P3 is a germline and belongs to the Q52 (VH2) gene family, which was previously observed in autoantibodies against gangliosides and frequently used by CD5^+^ B-1a lymphocytes [3, 4]. P3 mAb triggers a strong anti-idiotypic response in the syngeneic model, BALB/c mice, even in the absence of adjuvant or carrier protein [5], which is not a commonly observed phenomenon [6]. This high anti-P3 idiotype antibody response might be part of a protection mechanism to block self-reactive antibodies. The mechanisms best known to regulate B cell responses include negative selection, receptor editing, anergy, and the requirement for appropriate costimulation [7–9]. However, anti-idiotypic responses have also been associated with regulation of autoimmunity. Several studies have shown the existence of physiologically established anti-idiotypic antibody responses, which are absent or diminished during the development of several autoimmune diseases, suggesting they contribute to the control of autoreactivity [10–12]. So far, the B cells involved in these antibody responses have not been identified. P3 mAb recognizes and activates B-1a cells in vitro, inducing the secretion of cytokines, fundamentally IFN-*γ* [13]. However, the capacity of this antibody to stimulate B-1a cells in vivo has not been tested, neither the participation of B-1a and/or B-2 cells in the anti-idiotypic response.

Despite the fact that P3 is administered only in phosphate-buffered saline (PBS), the antibodies induced in BALB/c mice are predominantly IgG. Also, P3 is not immunogenic in C57BL/6 mice [14], which suggests it induces a MHC-restricted T cell-dependent anti-idiotypic response. Furthermore, previous data published by our group demonstrated that T lymphocytes obtained from the lymph nodes of BALB/c mice immunized with P3 mAb proliferated in vitro, in a dose-dependent manner, not only in the presence of P3 mAb but also in the presence of 1E10 mAb, an anti-P3 anti-idiotypic mAb. This result suggested that P3 mAb is able to activate an idiotypic cascade that involves B and T cells (B-T idiotypic cascade) [15]. Interestingly, it was also shown that the depletion of CD8^+^ T cells, before the first immunization with P3 mAb in PBS, inhibited the induction of anti-P3 antibodies [14]. Yet, how P3 activates CD8^+^ T cells and the physiological role of this activation have not been studied. It has been proven that not only B cells but also T cells can recognize both B and T cell variable region peptides and establish idiotypic interactions [16–25]. Many studies have demonstrated the participation of CD4^+^ T cells in B-T idiotypic interactions, especially those related to immune regulation [16, 17, 19–24]. However, idiopeptides derived from the heavy and/or light chains of anti-idiotypic antibodies can also be presented by MHC class I antigens to CTL, which has the capacity to stimulate or inhibit B cell responses [26–31]. Anti-idiotypic B cells can present idiopeptides carried by anti-self antibodies to T cytotoxic cells thus priming them in the absence of the antigen to kill the B cells presenting such idiopeptides [32–34].

The present work aims to understand the relevance of the unusual high response against a self antibody and to identify the B cells participating in this response. Our results show that both B-1a and B-2 cells are necessary to induce the anti-P3 idiotype IgG response. Although P3 mAb activated both B-1a [13] and B-2 cells in vitro, only B-1a cell activation was detected in vivo. B-2 cells from P3 mAb-immunized mice were able to activate naïve CD8^+^ T cells when they were restimulated in vitro with P3 mAb, while B-1a cells were able to do so even without adding P3 to the culture. This work also demonstrates for the first time the ability of P3 mAb idiotype to induce a specific cytotoxic CD8^+^ T cell response in vivo against cells expressing the idiotype. This study could help to elucidate alternative mechanisms to keep the tolerance against self-antigens under physiological conditions, through idiotypic interactions between B and T cells.

## 2. Materials and Methods

### 2.1. Animals

BALB/c and BALB/Xid mice, 6–8 weeks old, were purchased from the Center for Laboratory Animal Production (CENPALAB, Havana, Cuba). The animals were housed and bred in a barrier-maintained room according to the guidelines stipulated by the Animal Subject Committee Reviews Board at the Center of Molecular Immunology (CIM). Animal studies were performed with the approval from the CIM's Institutional Animal Care and Use Committee.

### 2.2. mAbs

P3 mAb (IgM, *k*) recognizes NeuGc-containing gangliosides and sulfated glycolipids [1]. P3 mAb was purified from ascitic fluid by gel filtration chromatography using a Sephacryl S-300 high-resolution column (Pharmacia) equilibrated with PBS containing 0.5 M NaCl. 6E4 mAb (IgM, *k*) anti-mouse EGFR which is not immunogenic in the syngeneic model was purified like P3 mAb and used as isotype control in all the experiments. E1 mAb (IgM, *k*), an anti-GM2 which is not immunogenic in the syngeneic model, was used as a control sequence for bioinformatics prediction of MHC class I-binding peptide.

### 2.3. Cell Isolation

Peritoneal B-1a cells were isolated using magnetic microbeads conjugated to a cocktail of biotin-conjugated antibodies against non-B-1a cells as first labeling reagent (B-1a Cell Biotin-Antibody Cocktail) and Anti-Biotin MicroBeads as secondary labeling reagent (Miltenyi Biotec), following the manufacturer's instructions. B-2 cells were isolated from mouse spleen using magnetic microbeads conjugated with rat anti-mouse B220 mAb (CD45R (B220) MicroBeads, Miltenyi Biotec) for in vitro assays or using magnetic microbeads conjugated to a cocktail of biotin-conjugated antibodies against non-B-2 cells as first labeling reagent (B Cell Isolation Kit; cocktail of biotin-conjugated antibodies against CD43 (Ly-48), CD4 (L3T4), and Ter-119) and Anti-Biotin MicroBeads as secondary labeling reagent (Miltenyi Biotec), for cell transfer assay, in both cases following the manufacturer's instructions. CD8^+^ T cells were isolated from mouse spleen using magnetic microbeads conjugated to a cocktail of biotin-conjugated antibodies against non-CD8^+^ T cells (CD8a^+^ T Cell Biotin-Antibody Cocktail; cocktail of biotin-conjugated monoclonal antibodies against CD4, CD11b, CD11c, CD19, CD45R (B220), CD49b (DX5), CD105, anti-MHC class II, and anti-Ter-119), as primary labeling reagent. Anti-Biotin MicroBeads were used as secondary labeling reagent (Miltenyi Biotec), following the manufacturer's instructions. The purity of cell populations was evaluated by flow cytometry; it was above 90% for B-1a and B-2 cells and above 85% for CD8^+^ T cells. Murine lymphocytes and hybridoma cells were cultured in RPMI-1640 medium (Gibco), supplemented with 10% fetal calf serum (FCS), sodium pyruvate, L-glutamine, 10 U/mL penicillin, and 10 mg/mL streptomycin (Gibco).

### 2.4. Induction of Antibody Response against P3 mAb

BALB/c and BALB/Xid mice were immunized with four biweekly subcutaneous doses of 50 *μ*g of P3 mAb or IgM isotype control in PBS. Serum samples were taken before the first and seven days after the fourth immunization. To determine the role of B cells in the induction of the antibody response against P3 mAb, B-1a peritoneal cells and B-2 splenic cells were magnetically purified as previously described in Section 2.3. 5 × 10^6^ B-1a cells, 50 × 10^6^ B-2 cells, or 5 × 10^6^ B-1a cells plus 50 × 10^6^ B-2 cells were transferred intraperitoneally to recipient BALB/Xid mice three days before the beginning of the immunization protocol. The number of B-1a [35, 36] and B-2 [36, 37] cells used in transference experiments was obtained from previous described protocols.

### 2.5. ELISA

Solid phase ELISA was performed as previously described [14] using 96-well polystyrene MaxiSorp microtiter plates (Nunc) coated with 10 *μ*g/mL of purified P3 mAb and incubated with preimmune or hyperimmune sera dilute 1 : 100. Alkaline phosphatase-conjugated goat anti-mouse IgG (Fc*γ*-specific) from the Jackson Immunoresearch Laboratories was used as a secondary antibody. To study the IgG subtypes of the anti-P3 mAb response, biotin-conjugated rat anti-mouse IgG1 (A85-1), IgG2a (R19-15), IgG2b (R12-3), or IgG3 (R40-82) (BD Biosciences) was used, followed by alkaline phosphatase-conjugated streptavidin (Sigma). The reaction was developed with *p*-nitro-phenyl phosphate substrate (Sigma) in dietanolamine buffer (pH 9.8). The absorbance was measured at 405 nm in an ELISA reader (iMark Microplate Reader, Biorad). For Ig quantification, plates were coated with 10 *μ*g/mL of goat anti-mouse IgM or IgG, incubated with serial dilutions of mouse serum. Alkaline phosphatase-conjugated goat anti-mouse IgM (Fc*μ*-specific; Jackson Immunoresearch) or IgG (Fc*γ*-specific) was used as secondary antibodies. To test the natural response against PPS3 in BALB/Xid mice, reconstituted with B-1a cells, MaxiSorp microtiter plates were coated with 5 *μ*g/mL of PPS3, overnight at 4°C. The quantification ELISA was performed as described above. Standard curves of purified mouse polyclonal IgM or IgG antibodies (Sigma, USA) were used in a range between 0.8 and 100 ng/mL for quantification purposes.

### 2.6. Recognition of B-2 Cell Subpopulation and Evaluation of B-2 Cell Activation In Vitro and In Vivo by P3 mAb

Splenic B-2 cells, isolated from naïve BALB/c mice, as described in Section 2.3, were incubated for 20 min on ice with 10 *μ*g/mL of biotinylated P3 mAb or control IgM followed by PECy5-conjugated streptavidin (BD Biosciences). With the aim to determine whether P3 mAb was able to activate B-2 cells in vitro, 3 × 10^5^ cells were isolated from naïve BALB/c mice and incubated for 72 h with 100 *μ*g/mL of P3 mAb or IgM isotype control. In order to study the capacity of P3 mAb to activate B-2 cells in vivo, B-2 cells were isolated from spleens of BALB/c mice immunized with a single dose of 50 *μ*g of P3 mAb. The expression of B cell activation markers was detected by flow cytometry by incubating the cells for 20 min on ice with anti-CD25/PE, anti-CD69 (M1.2F3)/PECy7, or anti-CD86/B7-2 (GL1)/PE while cell populations were identified using anti-B220/FITC and anti-CD5/PECy5 (BD Biosciences). The cytokine production was detected by intracellular staining. The cells were fixed and permeabilized, according to the manufacturer's protocol, with BD Cytofix/Cytoperm Buffers from BD Biosciences and incubated with anti-IFN-*γ*/PE, anti-IL-4/PE, and anti-IL-10/PE (BD Biosciences). To stablish the threshold of autofluorescent cells for each activation marker, control cells were stained with B220/FITC and anti-CD5/PECy5 plus the corresponding isotype controls (IC). In all the studies, the staining was detected using a Gallios analyzer flow cytometer and analyzed by the Kaluza 1.2 software (Beckman Coulter).

### 2.7. B-1 Cell Activation In Vivo by P3 mAb

In order to study the capacity of P3 mAb to activate B-1 cells in vivo, B-1 cells were isolated from peritoneal cavity washout, as described in Section 2.3, from BALB/c mice immunized with a single dose of 50 *μ*g of P3 mAb. The expression of B cell activation markers and cytokine production was detected as described in Section 2.6 above. The staining was studied using a Gallios analyzer flow cytometer and analyzed by the Kaluza 1.2 software (Beckman Coulter).

### 2.8. In Vitro Activation of CD8^+^ T Cells

BALB/c mice were immunized once, subcutaneously, with 50 *μ*g of P3 mAb or IgM isotype control in PBS. Three days after the immunization, the mice were sacrificed and peritoneal and splenic cells were collected. Peritoneal B-1a cells (B220^+^CD5^+^), splenic B2 cells (B220^+^CD5^−^), and splenic T CD8^+^ cells were magnetically purified as previously described in Section 2.3, following the manufacturer's guidelines. 1 × 10^5^ CD8^+^ T cells were cocultured with 2 × 10^5^ B cells (1 : 2) and incubated for three days with 100 *μ*g/mL of P3 mAb or IgM isotype control. The expression of activation markers and cytokine production by CD8^+^ T cells was evaluated by flow cytometry by incubating the cells for 20 min on ice with anti-CD25/PE, anti-CD69 (M1.2F3)/PECy7, or anti-CD107a/PE while the cell populations were identified by anti-CD8a/FITC or anti-CD8b/PE (BD Biosciences). The cytokine production and granzyme B expression was detected by intracellular staining. The cells were fixed and permeabilized, according to the manufacturer's protocol, with BD Cytofix/Cytoperm Buffers from BD Biosciences and incubated with anti-IFN-*γ*/PE and anti-Granzyme B/FITC (BD Biosciences). To stablish the threshold of autofluorescent cells for each activation marker, control cells were stained with anti-CD8a/FITC or anti-CD8b/PE plus the corresponding isotype controls (IC).

### 2.9. MHC Class I Molecule-Binding Peptide Prediction

The prediction of peptides, from P3 mAb V_H_ sequence, with the potential to bind to MHC class I molecules, was performed on February 2, 2013 using the IEDB analysis resource consensus tool [38], which combines predictions from ANN [39, 40], SMM [41], and comblib [42]. The selection criteria established were as follows: the peptide had an IC_50_ below 500 nM [43] and was not present in the variable region of a control IgM anti-ganglioside antibody, which is not immunogenic in the syngeneic model. To determine the specificity of the predicted peptide of P3 mAb, its expression was evaluated in all the heavy chain sequences of Abysis database, which compiles all the antibody sequences contained in IMGT, Kabat, and PDB databases. The sequences were downloaded in FASTA format and filtered to eliminate redundancy and the existence of uncertainties (deleted all sequences that have the residues X or B). The motives were analyzed with Linux *grep* command. At the moment of the analysis (April 2013), Abysis contained 8538 sequences, of which 7176 were nonredundant and 6843 had no sequence uncertainties. The prediction of MHC class I molecule ligands and the analysis of the motives were made with 6843 passing filters. The P3 mAb sequence appears twice in the database, as CS616230 and CS558783. Only CS616230 was used for the analysis. Afterwards, the predicted peptide of P3 mAb variable region was synthesized at the Center for Genetic Engineering and Biotechnology (Havana, Cuba).

### 2.10. CTL In Vivo Assays

The capacity of P3 mAb peptide to induce CTL in vivo was performed as previously described by Oehen and Brduscha-Riem [44] with slight modifications. Briefly, BALB/c mice were immunized with a single subcutaneous dose of 50 *μ*g of P3 mAb, IgM isotype control, or PBS. Seven days after the immunization, splenocytes from naïve mice were differentially labeled with CFSE, as previously described, 5 min at 37°C. The cells stained with CFSE_high_ (5 *μ*M) were used as targets and pulsed with P3 V_H_ peptide (3 *μ*M; 60 min at 37°C and 5% CO_2_), whereas the cells labeled with CFSE_low_ (0.33 *μ*M) were kept unpulsed and served as internal control. After extensive washing to remove free peptide, target and control cells were mixed at 1 : 1 proportion and coinjected i.v. into vaccinated mice. After 20 h, the spleens of recipient mice were harvested and the total events (#*Φ*) corresponding to both fluorescence intensity (CFSE_low_ and CFSE_high_) were determined by flow cytometry. In both experiments, the percentage of lysis was calculated using the following equation:

(1) $$\begin{array}{c} \%\;lysis=\left(1-\frac{\#\Phi {CFSE}_{high}Ab\text{.}/\#\Phi {CFSE}_{low}Ab.}{\#\Phi {CFSE}_{high}PBS/\#\Phi {CFSE}_{low}PBS}\right)*100. \end{array}$$

### 2.11. Statistical Analysis

Each experiment was performed at least twice. The differences between two groups were evaluated by the Mann–Whitney *U* test. The comparisons between more than two groups were performed by the Kruskall-Wallis followed by the Games-Howell or Duncan post-test. The differences were considered significant when *p* ≤ 0.05. All statistical tests were one-tailed and conducted using SSPS for Windows version 19.0.0.1 software.

## 3. Results

### 3.1. Evaluation of P3 mAb Ability to Activate B-1a Cells and Humoral Response to P3 mAb in BALB/Xid Mice

**Figure 1:**
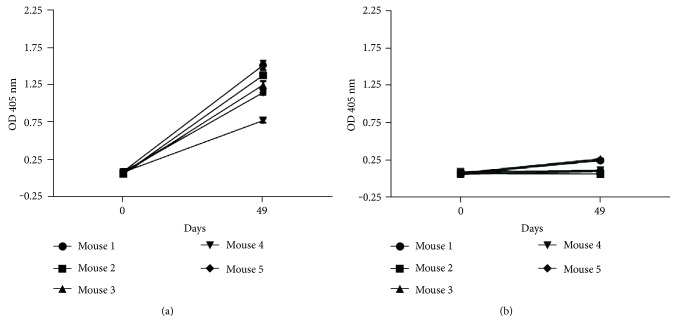
Antibody response against P3 mAb is abrogated in BALB/Xid mice. BALB/c (a) and BALB/Xid (b) mice were immunized subcutaneously with four doses of P3 mAb in PBS every two weeks. Serum samples were taken before the first immunization (day 0) and after the fourth dose (day 49). The kinetic of the response was determined by ELISA, on plates coated with P3 mAb, using alkaline phosphatase-conjugated goat anti-mouse IgG (Fc*γ*-specific). Sera were diluted to 1 : 100. The graphs show means ± SD of the values obtained in triplicate for each individual mouse.

P3 mAb administered only in PBS has the ability to generate a humoral immune response in the syngeneic BALB/c model [14]. Both P3 and its anti-idiotype 1E10 have V_H_ chains encoded by germline genes and the P3 V_H_, Q52, is highly represented in the fetal B-1a cell repertoire. In fact, previously we proved P3 is able to recognize and activate B-1a cells in vitro [13]. We wanted to establish whether B-1a cells participate in the idiotypic response against P3 mAb. With this purpose, BALB/c and BALB/Xid mice, which lack detectable numbers of peritoneal B-1a cells [45–47], were immunized with four biweekly doses of P3 mAb or an IgM isotype control. Although we detected by ELISA an antibody response against P3 mAb in all immunized BALB/c animals, no antibody response was detected after the four doses of P3 mAb in BALB/Xid mice (Figure 1), which suggests B-1a cells participate in the antibody response against P3.

**Figure 2:**
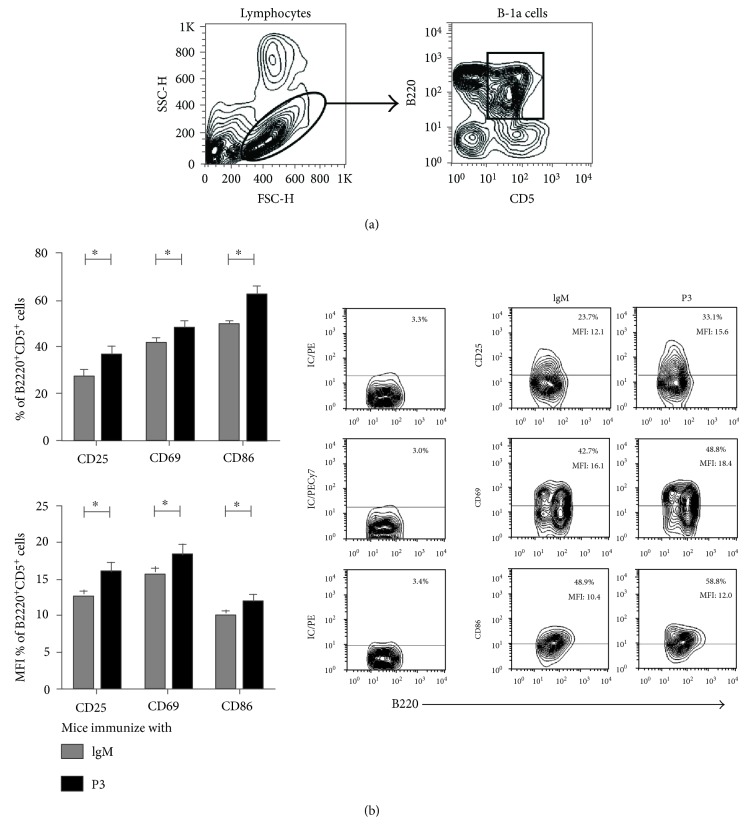
P3 mAb has the capacity to activate in vivo B-1a cells. BALB/c peritoneal B-1a cells were collected 72 h after the immunization with a single dose of 50 *μ*g of P3 mAb. (a) B-1a cell (B220^+^CD5^+^) gating strategy. (b) The ex vivo expression of CD25, CD69, and CD86 was evaluated by flow cytometry. Contour graph shows the results obtained for a representative mouse. Columns represent means ± SD of the values obtained in triplicate, ^∗^*p* < 0.05, Mann–Whitney *U* test.

Next, we tested whether P3 mAb was able to induce the activation of B-1a cells in vivo. We tested the expression of activation markers, CD25, CD69, and CD86, on the surface of B-1a cells directly ex vivo, three days after a single dose of P3 mAb. We observed an increase in the percentage of B-1a cells expressing all the markers tested, in BALB/c mice immunized with P3 mAb in comparison with B-1a cells derived from mice immunized with the control antibody (Figure 2).

### 3.2. Antibody Response against P3 mAb in BALB/Xid Mice Reconstituted with B Cells

**Figure 3:**
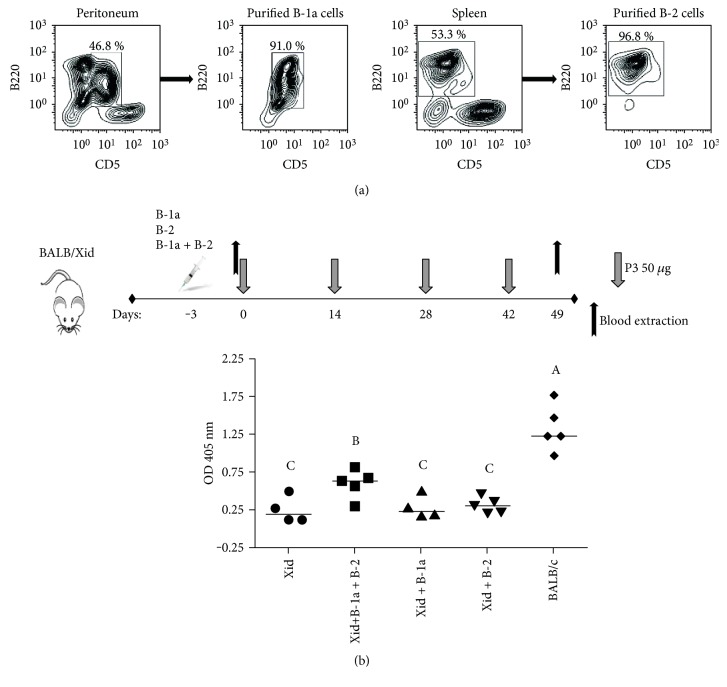
BALB/Xid mice transferred with B-1a plus B-2 cells recover IgG anti-P3 response. (a) Contour graph shows the results of B-1a and B-2 cell purification. (b) 5 × 10^6^ B-1a cells, 50 × 10^6^ B-2 cells, or 5 × 10^6^ B-1a cells plus 50 × 10^6^ B-2 cells from naïve BALB/c mice were transferred to BALB/Xid mice peritoneum, and mice were immunized subcutaneously with four doses of P3 mAb in PBS every two weeks. Serum samples were taken before the first immunization (day 0) and after the fourth dose (day 49). The reactivity was determined by ELISA on plates coated with P3 mAb using alkaline phosphatase-conjugated goat anti-mouse IgG (Fc*γ*-specific). Sera were diluted to 1 : 100. Symbols represent the median of the values obtained in triplicate for each individual mouse, *p* < 0.05, Duncan test. Different letters indicate statistical difference between the groups.

Since P3 mAb did not generate an antibody response in BALB/Xid mice, we evaluated whether the immunogenicity could be restored by transferring B-1a cells from BALB/c mice. After reconstitution with 5 × 10^6^ peritoneal B-1a cells, BALB/Xid mice were immunized with four doses of P3 mAb. The cell transfer was successful, since the numbers of B-1a cells in the peritoneal cavity increase three days after transference and the cells were functional, as measured by the recovery of the natural response against the B-1a antigen PPS3, at the end of immunization protocol (52 days after reconstitution) (see Supplementary Figure 1A available online at https://doi.org/10.1155/2017/2860867). Surprisingly, BALB/Xid mice reconstituted with B-1a cells did not elicit the IgG response against P3 mAb (Figure 3(b)), suggesting other B cells were also necessary. Then, we transfer 50 × 10^6^ splenic B-2 cells or a mix of 5 × 10^6^ peritoneal B-1a cells plus 50 × 10^6^ splenic B-2 cells to BALB/Xid mice and immunized them with four doses of P3 mAb. After three days, the total number of B-2 cells increased significantly in the peritoneal cavity but not in the spleen of the transferred mice, which can be explained due to a homeostatic regulation, since BALB/Xid mice have a significantly high number of B cells in the spleen. On the other hand, there was a turnover of B-2 population, since the levels of sera IgG show a recovery in mice transferred with B-2 cells at the end of immunization protocol (52 days after reconstitution) (Supplementary Figure 1B). Animals that received only B-2 cells also did not elicit an IgG response against P3 mAb. However, when we transferred both populations, B-1a and B-2 cells, we observed a significant IgG response against P3 mAb (Figure 3(b)). This result suggests that both B cell populations are participating in the anti-P3 mAb idiotypic response.

### 3.3. B-2 Cell Recognition and In Vitro Activation by P3 mAb

**Figure 4:**
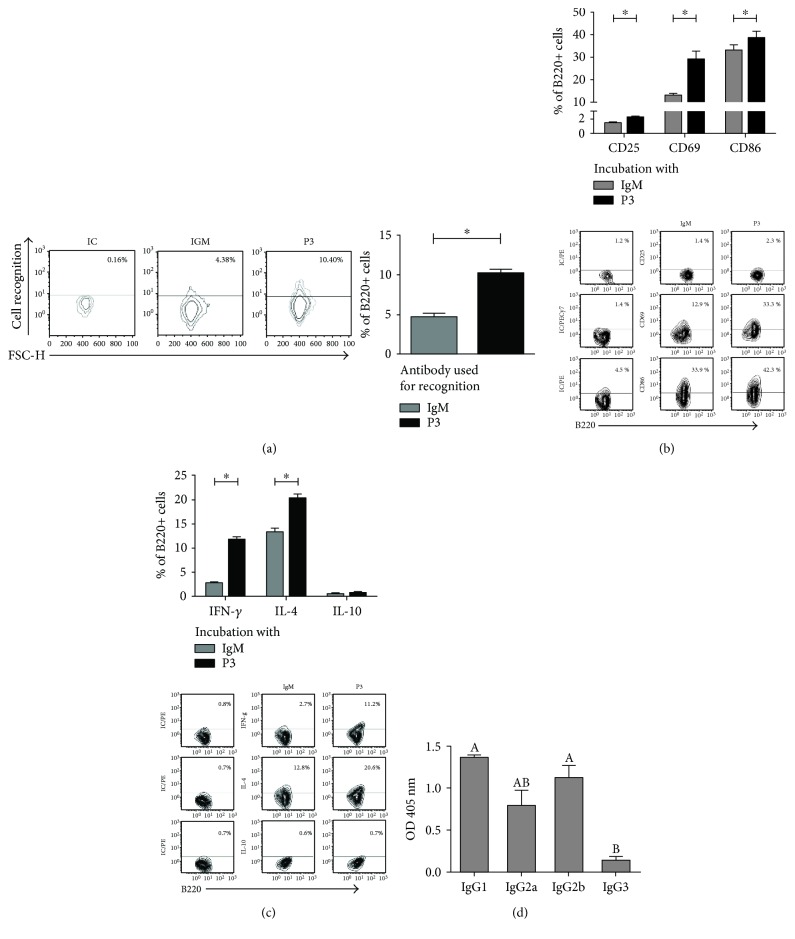
P3 mAb was able to recognize and activate in vitro B-2 cells. (a) Splenic B-2 cells isolated from naïve BALB/c mice were incubated with biotinylated P3 or control IgM mAbs, and the binding was evaluated by flow cytometry using a PECy5-conjugated streptavidin. Contour plots (left) show the results obtained for a representative mouse; columns (right) represent means ± SD of the values obtained for three mice. (b-c) Splenic B-2 cells obtained from naïve BALB/c mice were incubated three days with 100 *μ*g/mL of P3 mAb or IgM isotype control. Expression of the activation markers CD25, CD69, and CD86 was measured by flow cytometry (b), and cytokine production (IFN-*γ*, IL-4, and IL-10) was measured by flow cytometry after an intracellular staining protocol (c). (d) The IgG subclasses of the response against P3 mAb were determined in mice immunized subcutaneously with four doses of P3 mAb in PBS every two weeks. Sera samples were taken after the fourth dose (day 49). Sera were diluted to 1 : 100, and the reactivity against murine P3 mAb was assessed by ELISA using biotin-conjugated rat anti-mouse IgG1, IgG2a, IgG2b, or IgG3, followed by alkaline phosphatase-conjugated streptavidin. Means ± SD of the values obtained in triplicate for each individual mouse are graphed, ^∗^*p* < 0.05, Mann–Whitney *U* test (a–c) or Games-Howell test (d), both one-tailed. Different letters indicate statistical difference among the groups (d).

Since B-2 cells are also necessary for the development of the anti-P3 IgG response, next, we evaluated the capacity of P3 mAb to recognize and stimulate splenic B-2 cells from naïve BALB/c mice. P3 mAb was able to recognize up to 6 percent of B-2 cells derived from the spleen of naïve mice (Figure 4(a)). Furthermore, the incubation of B-2 cells with P3 mAb in vitro induced an increase of the activation markers CD25, CD69, and CD86 (Figure 4(b)). In addition, IFN-*γ* and IL-4 production increased, but not IL-10 (Figure 4(c)) or IgM secretion (data not shown). Consistent with the mixed IFN-*γ*/IL-4 cytokine profile of B-2 cells activated with P3 mAb, the anti-P3 mAb IgG response induced in BALB/c mice was of both IgG1 and IgG2a, b subtypes. IgG3 was not detected (Figure 4(d)). However, in contrast with B-1a cells, when the activation of B-2 cells was tested directly ex vivo, three days after a single dose of P3 mAb, we did not observe an increase in the expression levels of CD25, CD69, or CD86 on splenic B-2 cells (data not shown).

### 3.4. Capacity of B-1a and B-2 Cells, from Mice Immunized with P3 mAb, to Mediate the Activation of CD8^+^ Cells In Vitro

**Figure 5:**
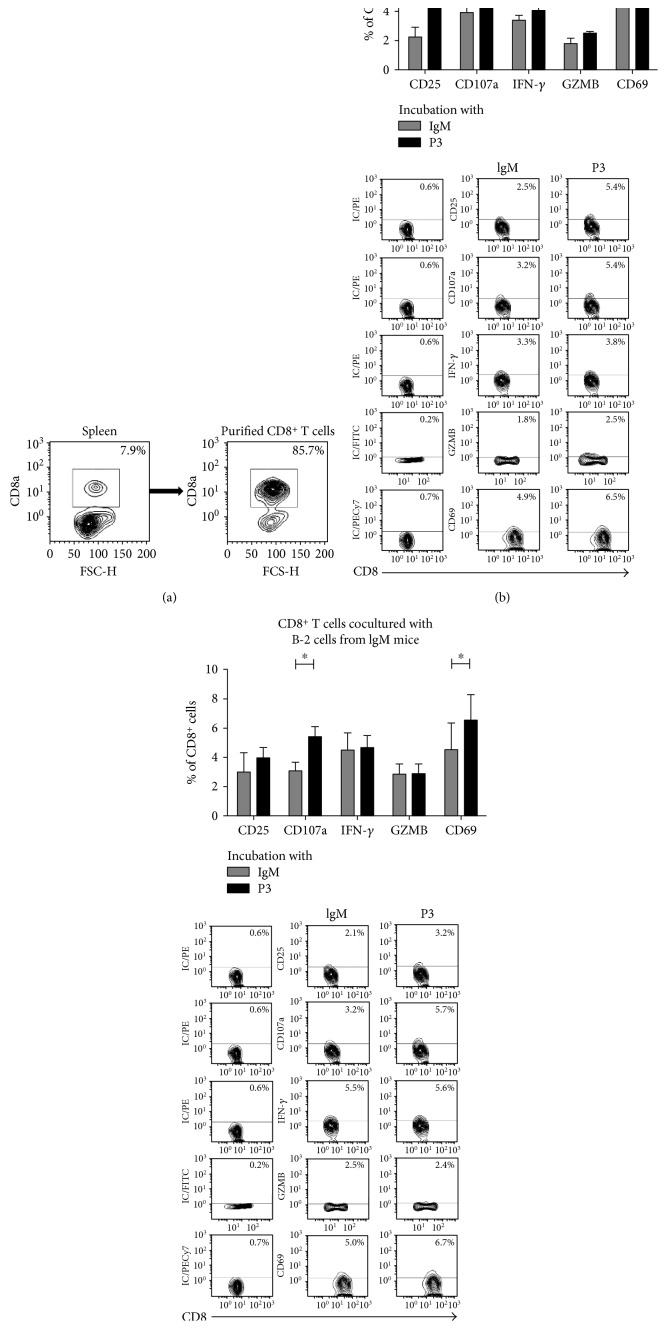
B-2 cells stimulated with P3 mAb were capable to induce in vitro CD8^+^ T cell activation. (a) Contour graphs represent efficiency of CD8^+^ T cell purification. Splenic B-2 cells derived from BALB/c mice immunized with a single dose of P3 mAb (b) or control IgM mAb (c) were incubated with naïve CD8^+^ T cells, and 100 *μ*g/mL of P3 mAb or control isotype antibody was added to the culture medium. After 72 h, the expression of activation and functional markers by CD8^+^ T cells was measured by flow cytometry, directly for CD25, CD107a, and CD69 or after an intracellular staining protocol for IFN-*γ* and granzyme B (GZMB). Contour plots show a representative result. Columns represent means ± SD of the values obtained in triplicate from a meta-analysis of two experiments, ^∗^*p* < 0.05, Mann–Whitney *U* test.

Previously, we demonstrated that CD8^+^ T cells are involved in the induction phase of the immune response against P3 mAb [14]. Herein, we tested whether B-1a and/or B-2 cells from P3 mAb-immunized BALB/c mice were able to activate naïve CD8^+^ T cells in vitro. Peritoneal B-1a and splenic B-2 cells isolated from mice that received one dose of P3 mAb were incubated with CD8^+^ T cells isolated from naïve mice, in the presence or not of P3 mAb or control IgM for 72 h. The expression of the activation and functional markers CD25, CD69, CD107a, and GZMB and IFN-*γ* secretion by CD8^+^ T cells were studied by flow cytometry. The coculture of splenic B-2 cells from P3-immunized mice with naïve CD8 T cells in the presence of P3 induced the activation of a significant percentage of CD8^+^ T cells, which overexpressed all the markers studied and secreted IFN-*γ* (Figure 5(a)). Even splenic B-2 cells from mice immunized with the control IgM, in the presence of P3 mAb, induced a significant increase of CD69 and CD107a positive CD8^+^ T cells (Figure 5(b)), confirming the capacity of P3 to activate B-2 cells in vitro. When P3 was not added to the culture, only an increase of CD25^+^CD8^+^ T cells was observed (data not shown). This result is in concordance with the fact that P3 immunization did not induce a detectable activation of B-2 cells in vivo, as showed above.

**Figure 6:**
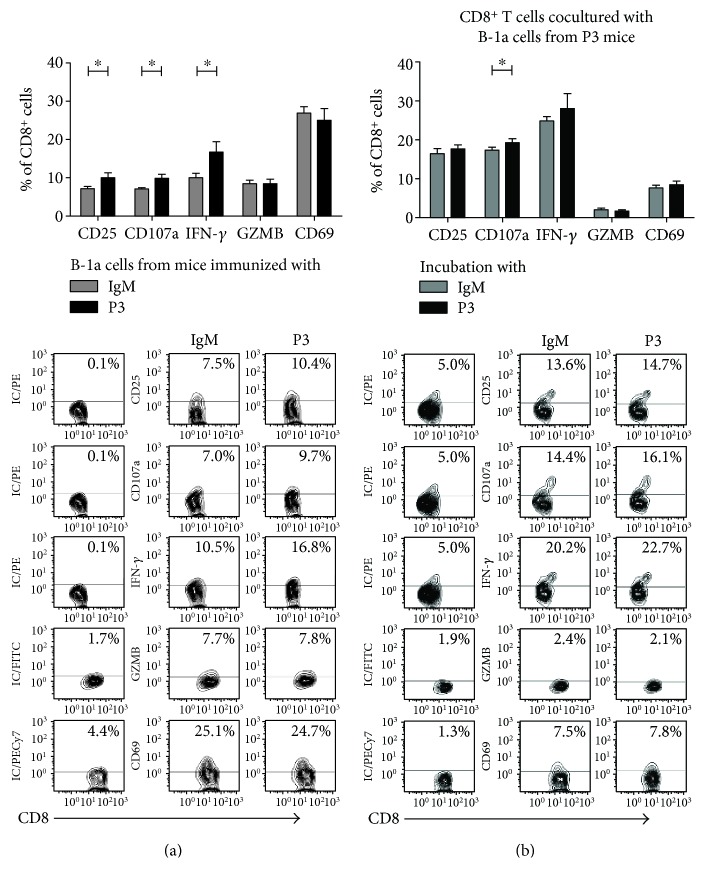
B-1a cells from P3 mAb-immunized BALB/c mice were capable to induce in vitro CD8^+^ T cell activation. (a) Peritoneal B-1a cells derived from BALB/c mice, immunized with a single dose of P3 or control IgM mAb, were incubated with naïve CD8^+^ T cells for three days. The expression of activation or functional markers by CD8^+^ T cells was measured by flow cytometry, directly for CD25, CD107a, and CD69 or after an intracellular staining protocol for IFN-*γ* and granzyme B (GZMB). (b) Peritoneal B-1a cells derived from BALB/c mice immunized with a single dose of P3 mAb were incubated with CD8^+^ T cells for three days, and 100 *μ*g/mL of P3 mAb or control isotype antibody was added to the culture medium. After 72 h, the expression of activation and functional markers by CD8^+^ T cells was measured as above. Contour plots show a representative result. Columns represent means ± SD of the values obtained in triplicate,^∗^*p* < 0.05, Mann–Whitney *U* test.

On the other hand, when naïve CD8^+^ T cells were incubated with B-1a cells derived from P3 mAb-immunized mice, the activation of a significant percentage of CD8^+^ T cells was detected, with increased CD25 and CD107a expression as well as IFN-*γ* production (Figure 6(a)). In contrast to the results obtained with B-2 cells, this activation was achieved regardless of the addition of P3 to the culture in vitro. The addition of P3 mAb to the culture had little effect over the activation of CD8^+^ T cells with only a slight increase of CD107a (Figure 6(b)). This result is in agreement with the significant activation detected ex vivo on B-1a cells isolated from P3-immunized mice. B-1a cells from control IgM-immunized mice did not induce CD8^+^ T cell activation, nor even when P3 mAb was added to the culture (data not shown). The percentages of activated CD8^+^ T cells detected in vitro were the same regardless whether CD8^+^ T cells were isolated from control or P3 mAb-immunized mice. The same results were obtained if instead of purified B cells whole spleen or peritoneal cells were used (results not shown).

### 3.5. Prediction of MHC Class I-Binding Peptide of P3 mAb Variable Region and Capacity of P3 mAb to Activate Specific CD8^+^ T Cell Cytotoxicity In Vivo

**Figure 7:**
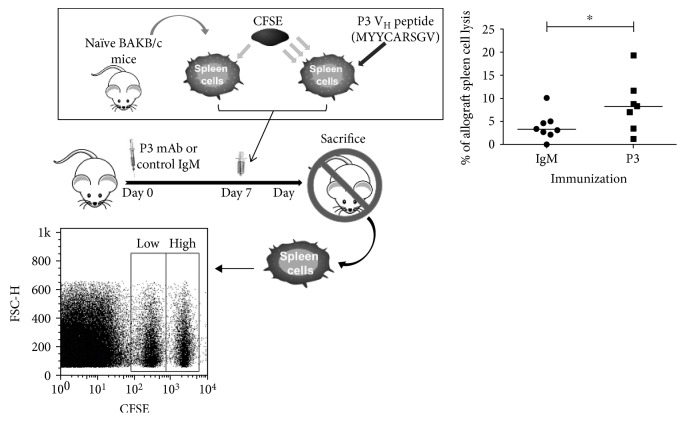
P3 mAb V_H_ region carries a peptide able to activate a CTL response after P3 mAb immunization. BALB/c mice were immunized with a single dose of 50 *μ*g of P3 mAb, IgM isotype control, or PBS, subcutaneously. Seven days after the immunization, each animal received, i.v., a 1 : 1 mixture of P3 V_H_ peptide-pulsed splenocytes labeled with a high concentration of CFSE (CFSE_high_) and unpulsed splenocytes labeled with a low concentration of CFSE (CFSE_low_). After 20 h, the spleens of the recipient mice were harvested and the total events corresponding to both fluorescence intensities (CFSE_low_ and CFSE_high_) were determined by flow cytometry. Each dot represents the percentage of specific lysis of P3 V_H_ peptide-pulsed splenocytes obtained for individual animals. The figure shows a meta-analysis combining three experiments. A representative dot plot graph shows the low and high CFSE-labeled populations. ^∗^*p* < 0.05, Mann–Whitney *U* test.

Since B cells activated with P3 mAb were able to activate CD8^+^ T cells, next, we tested whether CD8^+^ T cells were able to specifically lyse cells presenting P3 mAb peptides on their MHC class I molecules. First, we identified the possible peptides in P3 mAb variable region with the potential to stimulate CD8^+^ T cells. For this purpose, we utilized the immune epitope database (IEDB) analysis resource consensus tool (http://www.iedb.org/), in order to identify a peptide able to bind BALB/c MHC class I alleles with an IC_50_ less than 500 nM. The peptide should be present in the variable region of P3 mAb but not in another, nonimmunogenic, anti-ganglioside IgM antibody used as a negative control. Only one peptide fitted the two conditions established a priori. The peptide had the sequence MYYCARSGV and it was located in the junction of FR3 and CDR3. To determine whether this peptide was specific to P3 mAb, its sequence was searched within the sequences of all antibodies in Abysis database. After analyzing 6843 sequences, the result showed that this peptide's exact sequence is unique to P3 mAb. A similar sequence motif [MIV]Y[YF]C[AT]RSGV was presented in five (0.07%) antibodies (codes AF276278, 024255, 027483, 027482, and 001716), four of which expressed the YYCARSGV sequence. Finally, we wanted to evaluate whether the predicted peptide was able to mediate an in vivo cytotoxic T lymphocyte response after P3 mAb immunization. We used a CTL in vivo assay where BALB/c mice immunized once with P3 mAb or the control IgM received splenic cells labeled with CFSE and loaded or not with the MYYCARSGV peptide. The result showed that P3 mAb immunization was able to generate a specific CTL response that significantly kills only the transferred splenic cells loaded with the specific peptide (Figure 7). Thus, P3 mAb is able to induce in vivo a specific CD8^+^ T cell cytotoxic response in the syngeneic model against an idiopeptide.

## 4. Discussion

P3 is a monoclonal antibody, generated in BALB/c mice [1], that has the unique ability to induce in a syngeneic model a strong anti-idiotypic response in the absence of adjuvant or carrier protein [5, 14], which is not a frequently observed phenomenon [6, 48–50]. P3 was originally selected due to its binding to *N*-glycolylated gangliosides, but it also recognizes other self-molecules like sulfatides [1]. In addition, it shares 100% nucleotide identity with A4ac mAb, an IgM isolated from a mouse with experimental allergic encephalomyelitis. A4ac mAb is able to bind myelin oligodendrocyte glycoprotein [2] and other autoantigens including gangliosides [51]. Furthermore, P3 H-CDR3 carries a motif of arginine residues [4], which is fundamental for P3 mAb specificity and idiotypic immunodominance [52], and is common among autoantibodies against nuclear antigens [53], phospholipids [54, 55], chromatin [56], and cardiolipin [57]. The strong anti-idiotypic response P3 mAb induces could be a result of its specificity. Several studies have shown the existence of physiological anti-idiotypic responses, which are absent or diminished during the development of several autoimmune diseases, suggesting they play important roles in the control of autoreactivity [10–12]. Studies about systemic lupus erythematosus, type I diabetes, Sjögren's syndrome, and neonatal lupus syndrome showed that high anti-idiotype levels were found during remission in patients with autoimmune diseases but were diminished during the acute, active phases of the disease [11, 58–62].

In order to elucidate which B cell populations were participating in the anti-P3 idiotype response, BALB/Xid mice were immunized with P3 mAb. No IgG response was detected against P3 mAb, pointing to an important role of B-1a cells in the idiotypic response induced by this antibody. In fact, P3 mAb was able to activate this population in vivo, increasing the percentage of B-1a cells overexpressing CD25, CD69, and CD86. Our previous study showed that P3 was able to recognize up to 19% percentage of B-1a cells. This high percentage suggests a clonotypic recognition, where several B-1a clones are involved, which can be explained by the presence of a regulatory idiotope on P3 variable region [15]. Furthermore, P3 mAb also activated B-1a cells in vitro, without the mediation of any other immune cell, suggesting this activation is a direct effect, due to an interaction between P3 and B-1a cells [13]. However, B-1a cells transferred alone to BALB/Xid mice were not able to elicit an anti-P3 IgG response. This response was induced only when BALB/Xid mice were reconstituted with both B-1a and B-2 cells, suggesting both B cells participate in the establishment of the response. It is known that BALB/Xid mutation not only blocks the development of peritoneal B-1a but also results in deficiencies in B-2 cells and a decreased number of peripheral mature B cells [63–71]. The IgG response against P3 mAb in BALB/Xid mice reconstituted with B-1a and B-2 cells did not reach the level of response seen in BALB/c mice, but it has been reported that reconstituted Xid mice show differences in immune response parameters compared with the unmutated background strain [72–76]. Thus, we evaluated whether P3 mAb was also able to activate B-2 cells. In fact, up to 6 percent of splenic B-2 cells were recognized by P3 mAb and increased the expression of the activation markers after in vitro stimulation with this antibody. P3 mAb stimulated naïve B-2 cells to secrete IFN-*γ* and IL-4, but not IL-10, generating a mixed Th1/Th2 pattern, in congruence with the mixed IgG subtypes detected in anti-P3 mAb response. However, this activation was not confirmed in vivo, since we could not detect an increase in the percentage of B-2 cell overexpressing the activation markers directly ex vivo, after P3 immunization.

It was demonstrated in the previous work that the immunization with P3 mAb induced not only B cell but also T cell activation [15, 77]. We showed that CD8^+^ T cells were required to generate the anti-P3 mAb IgG response in BALB/c mice, suggesting an important role for these cells at the first stages of the anti-idiotypic response [14]. Several studies have proven that idiopeptides derived from antibodies are able to induce CD8^+^ cytolytic activity with the capacity to stimulate or inhibit B cell responses [26–31]. Some reports have shown CD8^+^ T cells can either directly activate B cells to secrete antibodies [30] or inhibit IgG production by suppressing T helper and B cell populations [31]. Herein, we demonstrate the capacity of P3 mAb to activate in vitro CD8^+^ T cells, in the presence of peritoneal B-1a or splenic B-2 cells. In both cases, CD8^+^ T cells showed an increase in activation markers, as well as a higher production of effector molecules and IFN-γ. However, there are differences in the capacity of these two B cell populations to activate CD8^+^ T cells. The activation of the B-1a cells in vivo by P3 mAb immunization was enough to enable B-1a cells to activate naïve CD8^+^ T cells in vitro. B-1a cells isolated from P3 mAb-immunized mice stimulated naïve CD8^+^ T cells, even without adding P3 mAb to the culture. These results suggest the activation levels achieved by B-1a cells in vivo, along with the fact that a high percentage of B-1a cells recognized by P3 mAb [13], are enough to activate CD8^+^ T cells in vitro. On the other hand, despite being isolated from P3-immunized mice, B-2 cells required further in vitro stimulation with this antibody in order to be able to properly activate CD8^+^ T cells. This difference with B-1a cells might be explained by two facts: first, there is a lower percentage of splenic B-2 cells recognized by P3 mAb, and therefore, their representation in the in vitro culture is much lower than that for B-1a cells. Second, contrary to B-1a cells, when we tested B-2 cell activation ex vivo, we did not see an increase in the expression of activation markers. Thus, B-2 cells need to be restimulated in vitro to induce CD8^+^ T cell activation. Based on the fact that B-2 cells need to be restimulated in vivo in order to activate CD8^+^ T cells, while B-1 cells can induce this activation directly ex vivo, without further stimulation, we hypothesize that B-1a cells should be the population presenting the idiopeptide to CD8^+^ T cells, in vivo, during the generation of the anti-P3 idiotypic response.

Next, we studied whether P3 was also able to activate cytotoxic CD8^+^ T cells in vivo, since it has been shown that CD8^+^ T cells can be activated by self-immunoglobulins and be cytotoxic against the B cell-secreting antibodies against a self-molecule [26, 78–80]. First, we looked for a peptide specific for the V_H_ region of P3 mAb. T cells are tolerant in a large extent to germline-encoded Ig V-region sequences [81], which is the case of P3 mAb V_H_. Usually, the available idiotype-specific T cell repertoire is largely confined to T cells that recognize rare idiopeptides due to somatic mutations or N region additions [81]. In our case, the predicted peptide in P3 mAb V_H_ was MYYCARSGV, located between FR3 and CDR3, a region very rich in N-additions [4]. This location differs from the one of a previously reported idiotope from an anti-idiotypic antibody able to activate CD8^+^ T cells, which was located in the NH2-terminal V_H_ region, before FR1 [82]. The peptide is unique to the P3 mAb since no other antibody from the database shares the exact sequence. A similar motif [MIV]Y[YF]C[AT]RSGV was present in only five of 6843 entries in the Abysis database. The existence of this particular MHC class I peptide in P3 mAb sequence could be one of the antibody's features that explain its high immunogenicity. A CTL assay, which measures the specific cytotoxicity of CD8^+^ T lymphocytes in vivo, demonstrated that the immunization with P3 mAb generated a specific CTL response against cells presenting the peptide.

**Figure 8:**
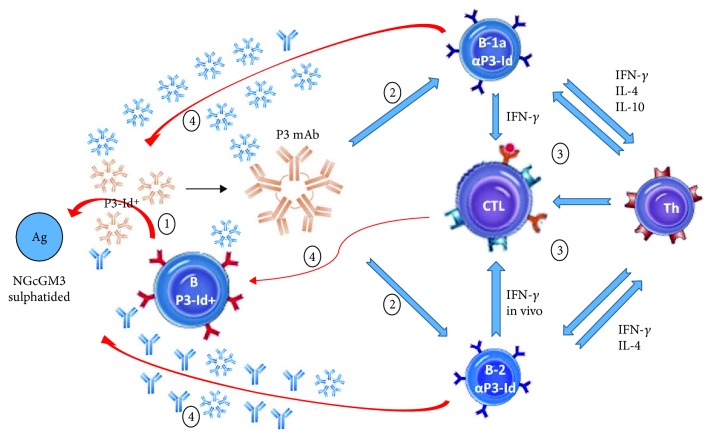
Model of peripheral tolerance to self-antigens mediated by anti-idiotypic responses involving P3 mAb. Different causes, like chronic infections, cross-reactivity between self and pathogen antigens, or dysfunction of regulatory circuits, can produce the proliferation and activation of B cell-secreting anti-self antibodies, similar to P3 mAb (P3-Id^+^) (1). Some of these antibodies (P3-Id^+^) carry a regulatory idiotope able to bind anti-idiotypic B cells, B-1a *α*P3-Id, and B-2 *α*P3-Id (2). These in vivo activated B-1a cells present idiopeptides and secrete cytokines that activate CD4^+^ (Th) and cytotoxic CD8^+^ T cells (CTL) (3). These activated cytotoxic CD8^+^ T cells can kill the anti-self B cells that present the regulatory peptide on their MHC class I, while the activated B cells, most likely B-2, will secrete anti-idiotypic IgG able to block the anti-self antibodies (4).

Several reports have shown the activation of suppressive idiotype-specific T cells in the response to self-antigens [83–86], suggesting an important role for the cytotoxic activity of CD8^+^ T cells, against anti-self antibody-secreting cells, in the control of autoimmunity. In fact, it has been demonstrated that idiotype-reactive T lymphocytes inhibit the development of autoimmune interstitial nephritis in rats [83], as well as anti-erythrocyte antibodies both in autoimmune NZB and in normal BALB/c mice [85, 86]. Interestingly, the antibodies with sequence homology to the peptide identified from P3 V_H_ region, codes 001716 [87] and AF276278 [88], were isolated from systemic lupus erythematosus-prone mice. It is possible that due to the anti-self-specificity, P3-like antibodies activate regulatory mechanisms including blocking of anti-idiotypic antibodies and the activation of cytotoxic T cells to eliminate the antibody-secreting cells recognizing self (Figure 8). This B-T idiotypic interaction and CTL activation could be an alternative mechanism to regulate responses against self-antigens under physiological conditions, which could be an especially relevant regulatory mechanism avoiding the response against self-antigens of nonprotein nature.

## Supplementary Material

Evaluation of B cell reconstitution and functionality after transfer to BALB/Xid mice.
