# Supplementary material for: B-CD8+ T Cell Interactions in the Anti-Idiotypic Response against a Self-Antibody
Source: J Immunol Res. 2017 Apr 9;2017:2860867. doi: 10.1155/2017/2860867 (PMC5401753; doi:10.1155/2017/2860867)
Supplement: Supplementary file 1 — Evaluation of B cell reconstitution and functionality after transfer to BALB/Xid mice. [file 2860867.f1.pdf]

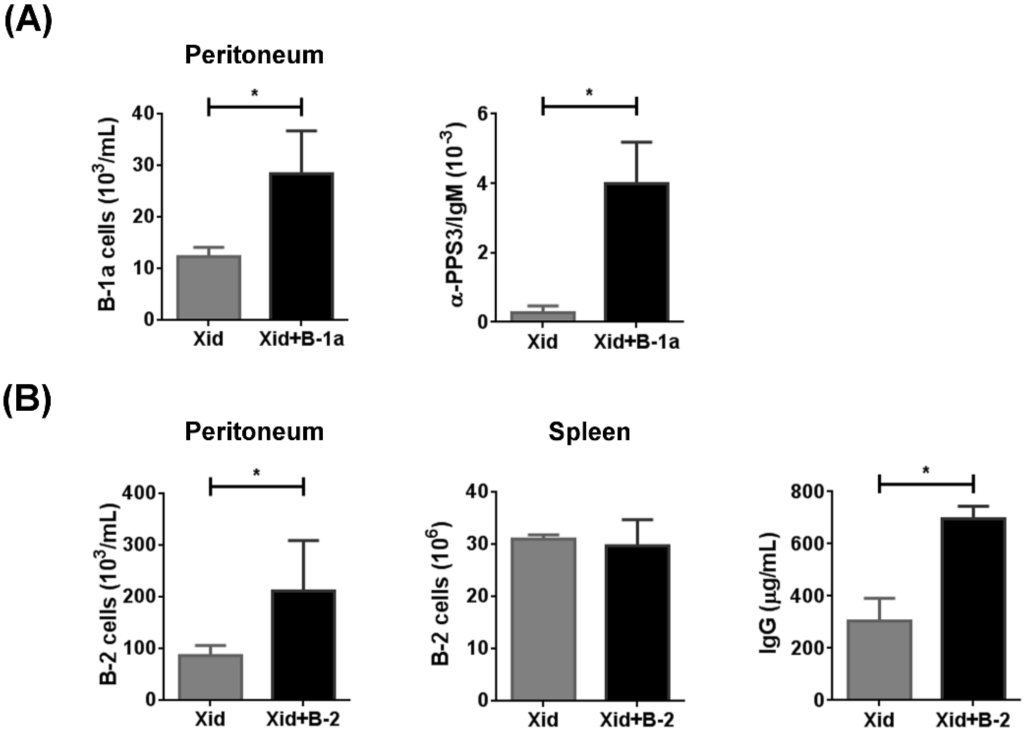

Supplementary Figure 1. Evaluation of B cell reconstitution and functionality after transfer to BALB/Xid mice. (A) Flow cytometer evaluation of the number of peritoneal B-1a cells and determination by ELISA of frequency of IgM anti-PPS3 antibodies among the total serum IgM pool after B-1a cells transfer to BALB/Xid mice. **(B)** Flow cytometer evaluation of the number of peritoneal and splenic B-2 cells, and determination by ELISA of total amount of IgG in mice serum after B-2 cells transfer to BALB/Xid mice. B cells population were measured three days after reconstitution while antibody responses were measured at the end of immunization protocol (52 days after reconstitution). Columns represent means ± SD of the values obtained in triplicate, * $p < 0.05$, Mann-Whitney U test.
